# Fournier’s Gangrene Secondary to Perforated Sigmoid Adenocarcinoma Within an Incarcerated Inguinal Hernia

**DOI:** 10.7759/cureus.49449

**Published:** 2023-11-26

**Authors:** Nicholas S Cairl, Felix Orelaru, Roy Golden

**Affiliations:** 1 General Surgery, Trinity Health Ann Arbor, Ann Arbor, USA; 2 Trauma, Acute and Critical Care Surgery, Trinity Health Ann Arbor, Ann Arbor, USA

**Keywords:** inguinal hernia, fournier's gangrene, sigmoid colon cancer, emergent general surgery, acute care surgery and trauma

## Abstract

Colon cancer is the third most common cancer worldwide. Approximately one-fifth of colon cancers will present emergently due to obstruction or perforation. Necrotizing soft tissue infection is a rare presentation of perforated colon cancer and represents a surgical emergency due to high mortality rate.

A man in his 80s presented with several days of scrotal pain and weakness. On physical exam he was found to have scrotal edema and erythema and bilateral inguinal hernias. Imaging revealed a large scrotal abscess and concern for necrotizing soft tissue infection. He was taken to the operating room for surgical debridement and exploration and was discovered to have perforated colon within an incarcerated inguinal hernia. He underwent exploratory laparotomy with sigmoid resection and end colostomy creation. Pathology returned demonstrating invasive sigmoid adenocarcinoma.

Fournier’s gangrene requires a high index of suspicion. It is a rapidly progressing infection associated with high mortality. Early initiation of antibiotics and surgical debridement are mainstays of treatment. When associated with perforated colonic malignancy, workup must include imaging of the chest, abdomen, and pelvis as well as carcinoembryonic antigen (CEA) level to complete staging.

Fournier’s gangrene secondary to perforated sigmoid adenocarcinoma is a unique presentation. Treatment first involves antibiotics and aggressive surgical debridement. Once the patient is stabilized, further oncologic workup should be completed to determine treatment course.

## Introduction

Colon cancer is the fourth most common cancer in the United States, accounting for 7.8% of all new cancer diagnoses [1]. It is the third most common cancer worldwide [2]. Up to 20% of colon cancers will present acutely as a surgical emergency related to perforation, obstruction, or bleeding [3-5]. Patients undergoing emergency surgery for colon cancer have been shown to have higher complication rates, peri-operative mortality, and decreased five-year survival rates compared to patients presenting electively [6,7].

Necrotizing soft tissue infection (NSTI), and more specifically necrotizing fasciitis, is a rare presentation of perforated colon cancer reported in the literature and is a surgical emergency in and of itself with a mortality rate between 25 and 30% [8]. Simonsen et al. analyzed a population-based database of insurance claims and found the incidence of necrotizing fasciitis to be 0.04 per 1000 person-years [9]. Necrotizing fasciitis requires a high index of suspicion and early and aggressive surgical debridement as delay in surgical intervention is associated with increased mortality [10].

Necrotizing fasciitis of the genitalia or perineal and perianal regions is referred to as Fournier’s gangrene [11]. Fournier’s gangrene is more common among males, with an incidence of 1.6 cases per 100,000 males per year [12]. It typically stems from trauma to the skin allowing for penetration of bacteria into the adjacent soft tissues [11]. Here, we present a unique case of Fournier’s gangrene secondary to perforated sigmoid adenocarcinoma within an inguinal hernia allowing for spread of colonic bacteria to adjacent perineal fascial planes resulting in necrotizing infection. 

## Case presentation

**Figure 1:**
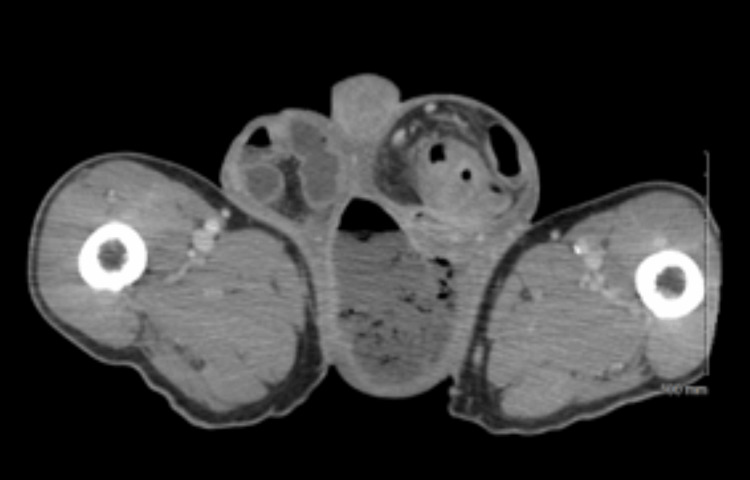
Axial imaging demonstrating bilateral inguinal hernias and a large fluid and gas collection in the left hemiscrotum

A man in his 80s presented to the emergency department with a three-day history of scrotal pain and generalized weakness. He had not seen a physician in 50 years and took no home medications. His initial vitals included a blood pressure of 105/51 mmHg, heart rate of 85 beats per minute, respiratory rate of 16 breaths per minute, and temperature of 37.1 degrees Celcius. On physical exam he was noted to have a benign abdomen and scrotal edema and erythema with palpable bowel within bilateral inguinal hernias. The right inguinal hernia was reducible; however, the left inguinal hernia was incarcerated. Laboratory studies were significant for a leukocytosis of 16.7 K/mcl, hemoglobin of 9.3 g/dL, sodium of 141 mEq/L, creatinine of 1.7 mg/dL, glucose of 87 mg/dL, and C-reactive protein (CRP) of 1.0 mg/dL. Imaging (Figure 1) demonstrated a 9.6 cm gas-containing fluid collection in the left hemiscrotum concerning for necrotizing soft tissue infection, a large left inguinal hernia containing 20 cm of sigmoid colon with a 4 cm segment of mural thickening, a right inguinal hernia containing small bowel, and multiple indeterminate liver lesions.

**Figure 2:**
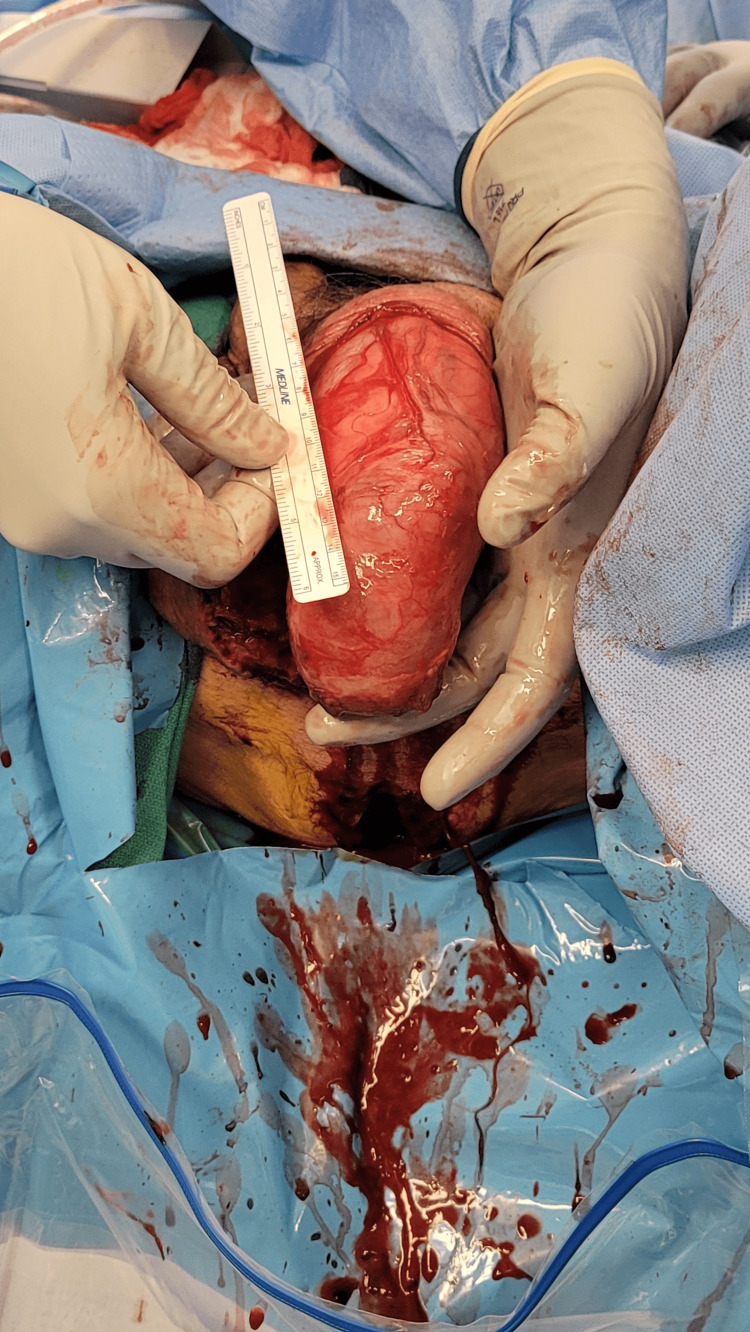
Intra-operative photo depicting colon within the left inguinal hernia

**Figure 3:**
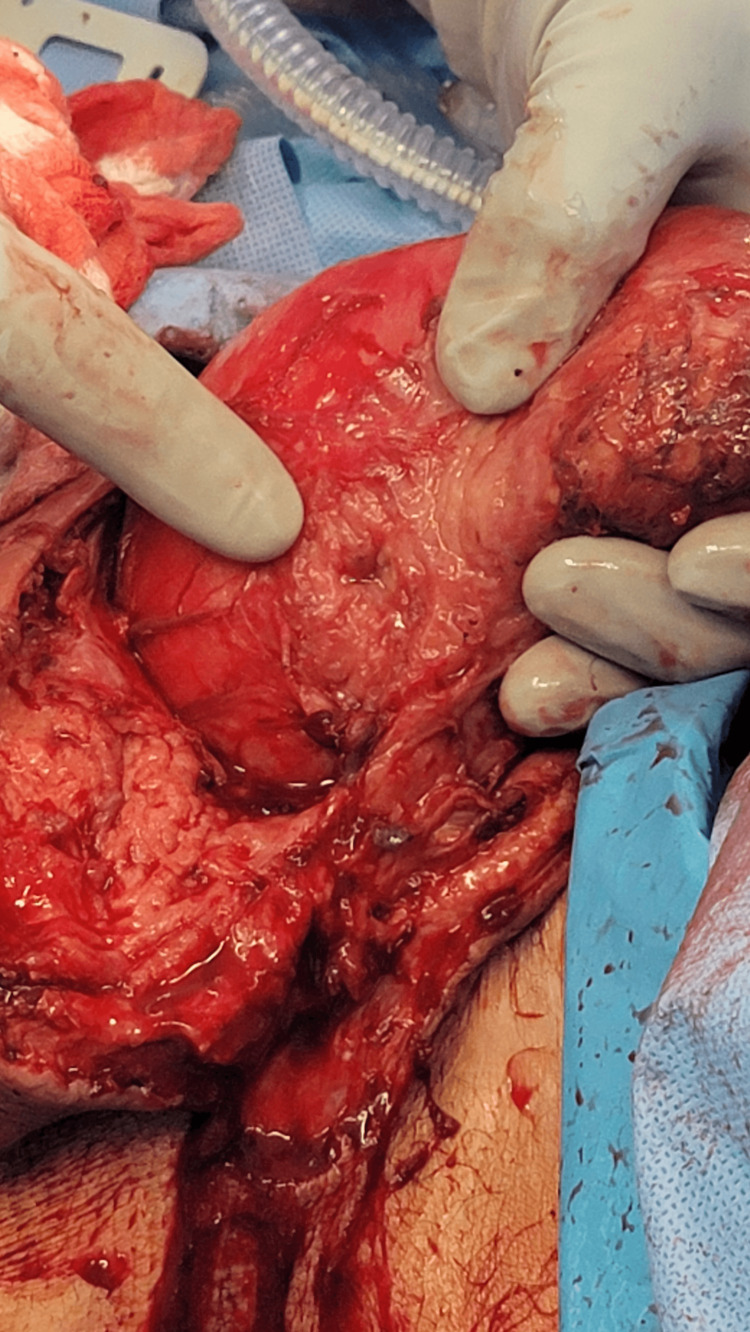
Intra-operative photo depicting macroscopic sigmoid perforation

The patient was started on broad-spectrum antibiotics including cefepime, vancomycin, and metronidazole, and initially underwent incision and drainage of his scrotal abscess in the emergency department with return of 200 cc of purulent fluid. The following day, due to worsening erythema, the patient was taken to the operating room where he underwent further debridement of his perineum, groin and scrotum with urology and general surgery. The left hemiscrotum was entered, exposing the patient’s colon (Figure 2). Upon exposing the colon, a macroscopic perforation was found (Figure 3). This was resected and the remainder of the herniated colon was then able to be reduced. A biologic mesh was placed in the left pelvis to prevent herniation of the reduced colon. Due to the significant infection and because the time under anesthesia was approaching five hours, it was felt that further time spent dissecting and reconstructing would be deleterious to the patient’s clinical status. A temporary abdominal closure device was placed and the patient was extubated and transported to the surgical intensive care unit (ICU). His vital signs and urine output were closely monitored and he was resuscitated as needed with crystalloid. Operative cultures grew *Klebsiella pneumoniae, Enterococcus faecalis, *and *Streptococcus lutetiensis.* He was taken back to the operating room three days later where he underwent left orchiectomy, reinforced biologic mesh placement, colostomy creation, and abdominal wall closure. He eventually underwent complex repair of his groin and scrotal wounds with plastic surgery. He was discharged to a subacute rehabilitation facility on post-operation day 10. 

**Figure 4:**
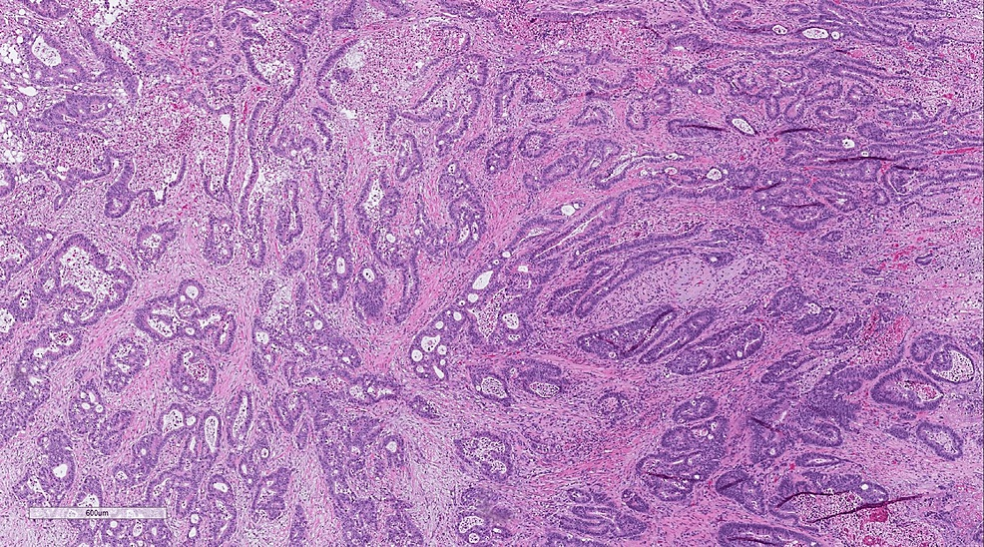
Histology demonstrating adenocarcinoma

**Figure 5:**
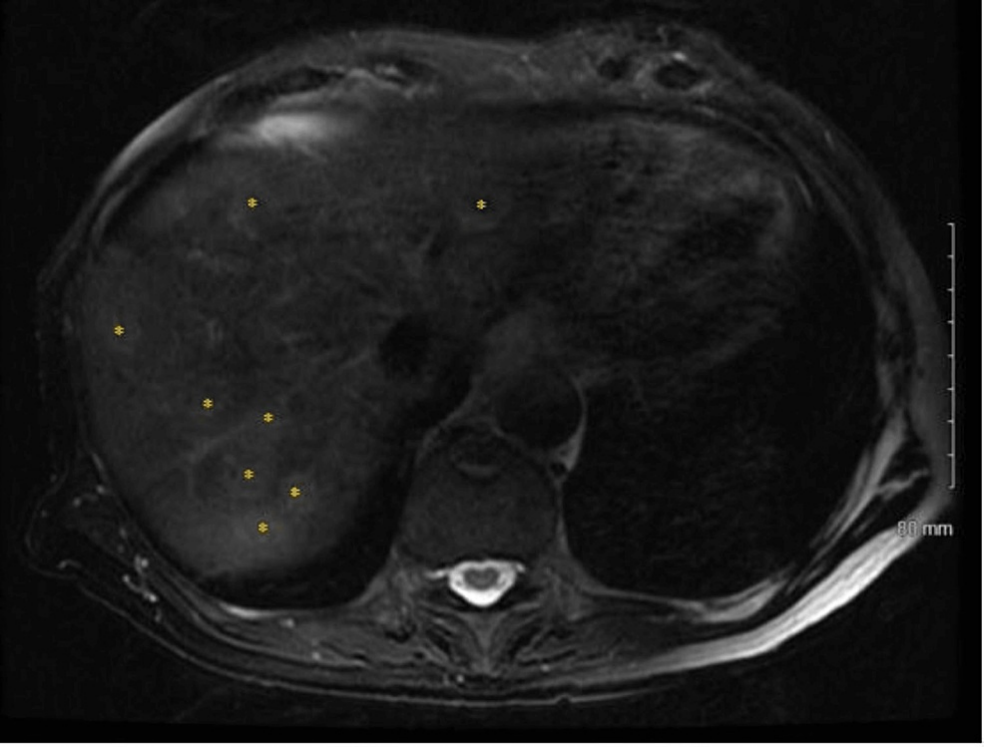
MR abdomen demonstrating hepatic metastases Hepatic metastases are denoted with an asterisk

Pathology demonstrated invasive adenocarcinoma of the sigmoid colon (Figure 4) with macroscopic perforation and 0 out of 16 lymph nodes involved, TNM stage pT4a pN0 pMx, mismatch-repair (MMR) proficient. All margins were uninvolved. Subsequent staging workup showed no evidence of disease in the chest and a carcinoembryonic antigen (CEA) level of 7.6 µg/L. Magnetic resonance imaging (MRI) of the abdomen demonstrated more than 15 hepatic masses consistent with metastases (Figure 5). The patient started adjuvant chemotherapy with capecitabine and was eventually lost to follow-up.

## Discussion

Patients with Fournier’s gangrene commonly present with scrotal swelling, pain, and erythema and are more likely to be obese and have diabetes [12,13]. On physical exam, erythema, edema, and crepitus may be present; however, external manifestations develop late in the disease course after a significant degree of internal necrosis has occurred [14,15]. While Fournier’s gangrene is a clinical diagnosis, imaging may provide additional information regarding the extent of the disease but should not delay operative intervention. Computed tomography (CT) is the most specific imaging modality for Fournier’s gangrene and may reveal fascial thickening, abscess, or subcutaneous emphysema [16]. The Laboratory Risk Indicator for Necrotizing Fasciitis (LRINEC) score is a clinical tool developed for early diagnosis of necrotizing fasciitis, including Fournier’s gangrene; however, it has been found to have poor sensitivity giving import to a high index of clinical suspicion [17].

Treatment of Fournier’s gangrene begins with resuscitation, including correction of electrolyte disturbances, and administration of broad-spectrum antibiotics. The mainstay of treatment, though, is surgical debridement. Debridement of all devitalized tissue should be performed until healthy, viable tissue is encountered. Re-exploration is often required 24-48 hours later, as repeated debridement is usually necessary [18]. In a retrospective review of patients with Fournier’s gangrene, Chawla et al. determined patients required an average of 3.5 debridements [19]. Due to the large skin defects created by debridement, patients typically require reconstruction once stable [11]. In this case, there was initially low suspicion by the emergency department for a necrotizing infection. The patient's LRINEC score on presentation was 5; however, as stated above, the LRINEC score is associated with poor sensitivity. While he did undergo early incision and drainage of his scrotal abscess, he likely would have benefitted from earlier surgical consultation and initial wide debridement. 

Treatment is complicated when the source of Fournier’s gangrene is perforated colon cancer and even further complicated when the perforated colon is contained within an incarcerated inguinal hernia. The basic tenets of resuscitation, broad-spectrum antibiotics, and surgical debridement still hold true; however, control of the perforation often requires laparotomy, reduction of the hernia, and colectomy. As in this case, despite a contaminated field, a biologic mesh can be used to repair the inguinal hernia to prevent re-herniation of abdominal contents into the debrided scrotum. If pathology returns positive for malignancy, a complete staging work-up should then be pursued, including CT or MRI of the chest, abdomen, and pelvis and CEA level. Synchronous malignancy should also be ruled out by colonoscopy, which can be done through the stoma in cases where colostomy is required.

## Conclusions

Fournier’s gangrene is an aggressive soft tissue infection with high mortality requiring high clinical index of suspicion. Fournier’s gangrene secondary to perforated sigmoid adenocarcinoma within an incarcerated inguinal hernia is a unique presentation and requires more complex treatment by a multidisciplinary team. Surgical intervention involves debridement of devitalized tissue and management of the colonic perforation and inguinal hernia. Once malignancy is identified and the patient has been stabilized, a full staging work-up should be completed and referral to oncology should be made to determine the course of treatment. 
